# A novel provocation protocol to identify nocebo effects in clinical practice—a proof-of-concept n-of-1 study

**DOI:** 10.3389/fphar.2026.1913605

**Published:** 2026-09-18

**Authors:** Martin Coenen, Gunther Hartmann, Thomas Büttner

**Affiliations:** Institute for Clinical Chemistry and Clinical Pharmacology, University Hospital Bonn, University of Bonn, Bonn, Germany

**Keywords:** adverse drug effects, alcohol, alcohol intolerance, drug intolerance, nocebo effect, nonspecific drug effects, provocation test

## Abstract

**Introduction:**

Nocebo effects, driven by negative expectations rather than pharmacological mechanisms, are an under-recognized cause of adverse effects in clinical practice. Although experimental studies have used expectation manipulation to explore these phenomena, translation into clinical diagnostic approaches remains limited. We report the first clinical application of a novel provocation protocol in a single patient as an n-of-1 trial to illustrate its potential to identify whether reported adverse events in this patient were driven by nocebo mechanisms.

**Methods:**

We developed an extension of the crossover balanced placebo design incorporating additional manipulation of patient expectations comprising five structured provocation sessions using an active substance or placebo combined with correct, and intentionally misleading declarations under open-label and blinded conditions. As a proof-of-concept this protocol was applied to a single patient with self-reported alcohol intolerance using ethanol and water. Symptom severity for each provocation was assessed using a numeric rating scale and analyzed descriptively.

**Results:**

The most severe symptoms followed the administration of open-label ethanol (mean numeric rating scale [NRS] 5.3; maximum 8) and water deceptively labeled as ethanol (mean NRS 4.1; maximum 7), while blinded ethanol (mean NRS 2.8; maximum 5) and blinded water (mean NRS 2.6, maximum 5) produced only mild reactions. These findings are consistent with adverse reactions in this single patient primarily driven by expectations, indicating a nocebo mechanism.

**Conclusion:**

This report demonstrates the proof-of-concept value of a novel “mixed open blinded crossover balanced placebo provocation protocol” and illustrates the potential to identify nocebo effects in clinical practice. Whether this exploratory finding have broader relevance warrants investigation in larger cohorts.

## 1 Introduction

Nocebo effects describe negative outcomes of medical treatments in clinical research or clinical practice that cannot be attributed to the drug’s genuine pharmacological action but rather result from negative expectations shaped by learning processes such as conditioning and contextual factors (Colloca and Barsky, 2020; Colloca, 2024). Although under-recognized in clinical practice, these effects present a major challenge in medical care as a well-documented barrier to effective therapy (Bingel, 2014; Burke et al., 2026), as a substantial proportion of reported drug-related adverse events may be attributed to nocebo responses (Rǎducan-Florea et al., 2024), especially in treatments with high media visibility or prior negative experiences (Barsky et al., 2002). However, nocebo effects are rarely systematically identified in routine clinical practice as no standard diagnostic approach exists. For the same reason, the frequency of the phenomenon can only be estimated from clinical trials, where it has been reported in 19% of persons taking placebos (Rosenzweig et al., 1993). During oral provocation testing nocebo reactions occur in 3% of patients (Barbaud et al., 2024). As established causality assessment instruments such as the Naranjo algorithm (Naranjo et al., 1981) or the WHO-UMC criteria (WHO, 2014) contain no item addressing patient expectations, so that a nocebo reaction fulfils the same criteria as a pharmacologically mediated one—onset after intake, resolution on withdrawal, recurrence on re-exposure—nocebo effects are often misclassified as probable adverse drug reaction and consequently as drug intolerance (O’Keeffe et al., 2026). This results in impaired adherence and increased discontinuation rates in a wide range of therapeutic settings. Our experience in our Clinical Pharmacology Outpatient Department shows that interventions are needed to better differentiate true drug intolerance from nocebo-driven adverse effects, particularly in patients who report intolerance to multiple substances, thereby enabling the continuation of essential therapies and improving patient care (Koulouridi et al., 2025). Open rechallenge, otherwise the most conclusive diagnostic step (Li and Kendall, 2001), is uninformative wherever an expectancy component is plausible, since the patient’s awareness of exposure is precisely the variable in question.

In a recently published case report, we describe a blinded crossover challenge in a patient with presumed statin intolerance in routine clinical practice (Büttner et al., 2025). This approach is based on the strategy of blinded challenge tests applied to patients with suspected adverse drug reactions (Liccardi et al., 2004; Lombardi et al., 2008). This case demonstrates that a simple structured single-blind placebo provocation test could be potentially diagnostically and therapeutically informative, as well as feasible in clinical care, for unmasking nocebo effects.

For an even clearer and more definitive diagnosis, additional active manipulation of patient expectations would be beneficial, an approach that has been frequently employed in studies under experimental conditions, primarily for investigations of the placebo effect (Enck et al., 2011; Lund et al., 2014). We therefore developed an extension of this method by applying the principles of the *balanced placebo design* (BPD) (Rohsenow and Marlatt, 1981). The classical BPD separates the pharmacological and psychological (expectancy-driven) components of a treatment by using four different conditions in a *parallel-group design*: (1) drug administered/told drug, (2) drug administered/told placebo, (3) placebo administered/told drug, and (4) placebo administered/told placebo. This 2 × 2 factorial structure allows separate estimation of the “true” pharmacological effect (condition 2 vs. condition 4) and the “true” expectancy or placebo effect (condition 3 vs. condition 4), as well as their potential interaction. The *modified crossover balanced placebo design* has the advantage of reducing interindividual variation and sample size by administering both placebo and verum sequentially within the same patient. In the crossover BPD described by Hammami et al. (2010), participants were randomized to *two groups*: one group received the active substance twice (once labeled as active substance, once as placebo), while the other group received placebo twice (once labeled as placebo, once as active substance), each in a randomized within-subject crossover design. An alternative crossover variant randomizes participants to *four groups* in which all are told they are taking part in a conventional double-blind crossover trial but only two groups actually alternate between active substance and placebo, while the remaining two groups receive either active substance or placebo exclusively on both occasions—allowing estimation of the expectancy component independently of the pharmacological effect (Enck et al., 2011). A third variant combines elements of both approaches in a hybrid of balanced placebo and randomized placebo-controlled designs, in which participants are randomized to three groups receiving active substance or placebo described as either active substance, placebo, or unknown in a crossover design, allowing simultaneous estimation of drug, placebo, and drug–placebo interaction effects (Hammami et al., 2016). Although these methodologies are well established in experimental studies (Rohsenow and Marlatt, 1981; Lund et al., 2014; Hammami et al., 2016), they have not been used in routine clinical diagnostics so far. However, the clinical assessment of nonspecific adverse drug reactions requires an adaptation that combines the various provocation conditions for use within the same subject—an approach not covered by existing designs. We therefore further developed these protocols into a five-phase crossover design incorporating open-label and blinded administrations of placebo and active substance adding correct and intentionally misleading information within the same individual to systematically manipulate patient expectations and thereby disentangle pharmacological from expectancy-driven components of adverse reactions. Whereas the individual components of our novel design are each known from placebo research, the distinctive feature lies in the systemic integration of the different elements, which opens up novel diagnostic possibilities in routine clinical practice: (1) the complete crossover of all provocation conditions *within the same patient*, rather than a parallel-group design across different patients or a crossover of only selected conditions; (2) the introduction of deceptive provocation into *this* crossover variant to explicitly manipulate expectations; (3) the *combination of blinded and open-label* provocations *within the same individual*.

We report the first clinical application of this novel provocation protocol for the diagnostic workup of a patient with self-reported alcohol intolerance, thereby demonstrating the initial feasibility of translating this approach into routine clinical practice in a single case, without providing evidence of diagnostic accuracy or generalizability. This translation of the systematic diagnostic workup of nocebo effects in clinical practice, has—to the best of our knowledge—rarely been investigated (Büttner et al., 2025) though frequently suspected in patients and therefore of great clinical relevance. The aim of this single case approach was to explore whether expectation manipulation can help differentiate pharmacological from psychological components of adverse reactions in clinical practice and should not be understood as an attempt to establish generalizability. The n-of-1 trial design—in which multiple treatment or exposure conditions are compared within the same individual to address an individualized clinical question—represents a methodologically appropriate framework for this diagnostic approach (Lillie et al., 2011; Qi, 2026). Since nocebo effects are by nature highly individual, depending on each patient’s expectations, prior experiences, and contextual factors, the relevant diagnostic question is inherently patient-specific. An n-of-1 design is therefore not a compromise but the method of choice, as it allows a direct, internally valid within-subject comparison in which the patient serves as his own control across all provocation conditions (Knowles et al., 2002). In contrast to other n-of-1 trials that have likewise been used to investigate adverse drug reactions, our design additionally manipulates patient expectations to disentangle pharmacological from expectancy-driven components of adverse reactions (Herrett et al., 2021).

## 2 Materials and equipment

The provocations require an active substance (verum) and a placebo. In the present application, 2 mL of 99% ethanol (hand disinfectant “Sterilium® med,” BODE Chemie GmbH, Hamburg, Germany) was used as the active substance and 2 mL of tap water as the placebo, both cooled to 4 °C prior to administration. Both substances were stored in identical dropper bottles bearing sequential numerical labels only (1–5), without any indication of their contents. A sealed decoding list, accessible to the administering clinician at all times, allowed unblinding at any point. For the administration procedure a sterile pipette, a FFP2 mask and a gauze compress were used.

The protocol is generally not restricted to provocations with ethanol; any active substance suspected of causing adverse reactions in an individual patient and a suitable inert placebo may be used. In many clinical scenarios, oral administration is the more relevant route, as the majority of medications are taken in the form of tablets or capsules. In such cases, an unlabelled tablet or capsule of the active substance and a commercially available placebo preparation that matches it as closely as possible in size, shape, and colour should be selected as described in our previously published statin provocation protocol (Büttner et al., 2025). A methodologically even more rigorous, albeit more resource-intensive alternative is to encapsulate both the active substance and an inert placebo in identical capsules, which requires the involvement of a compounding pharmacy. For other routes of administration, such as injectable or dermal formulations, the procedure would need to be adapted accordingly.

All provocations should be conducted in the same dedicated clinical room under standardized ambient conditions, including neutral lighting and consistent room temperature if possible, to minimize contextual variability between sessions. Furthermore, provocations should wherever possible be scheduled at the same time of day.

For practical reasons, symptom documentation is simplified using a structured symptom diary—paper-based or digital—in which symptom severity is rated daily using a standard numeric rating scale (NRS; 0–10).

## 3 Methods

### 3.1 Provocation protocol

We developed a provocation protocol (*Bonn Provocation Protocol*) consisting of five provocation sessions each involving a single application (Figure 1A):

- Provocation 1: Open-label administration of placebo, correctly declared (negative control)
- Provocation 2: Single-blind administration of placebo (patient unaware of content)
- Provocation 3: Deceptively-labeled administration of placebo declared as active substance
- Provocation 4: Single-blind administration of the active substance (patient unaware of content)
- Provocation 5: Open-label administration of the active substance, correctly declared (positive control).

**FIGURE 1:**
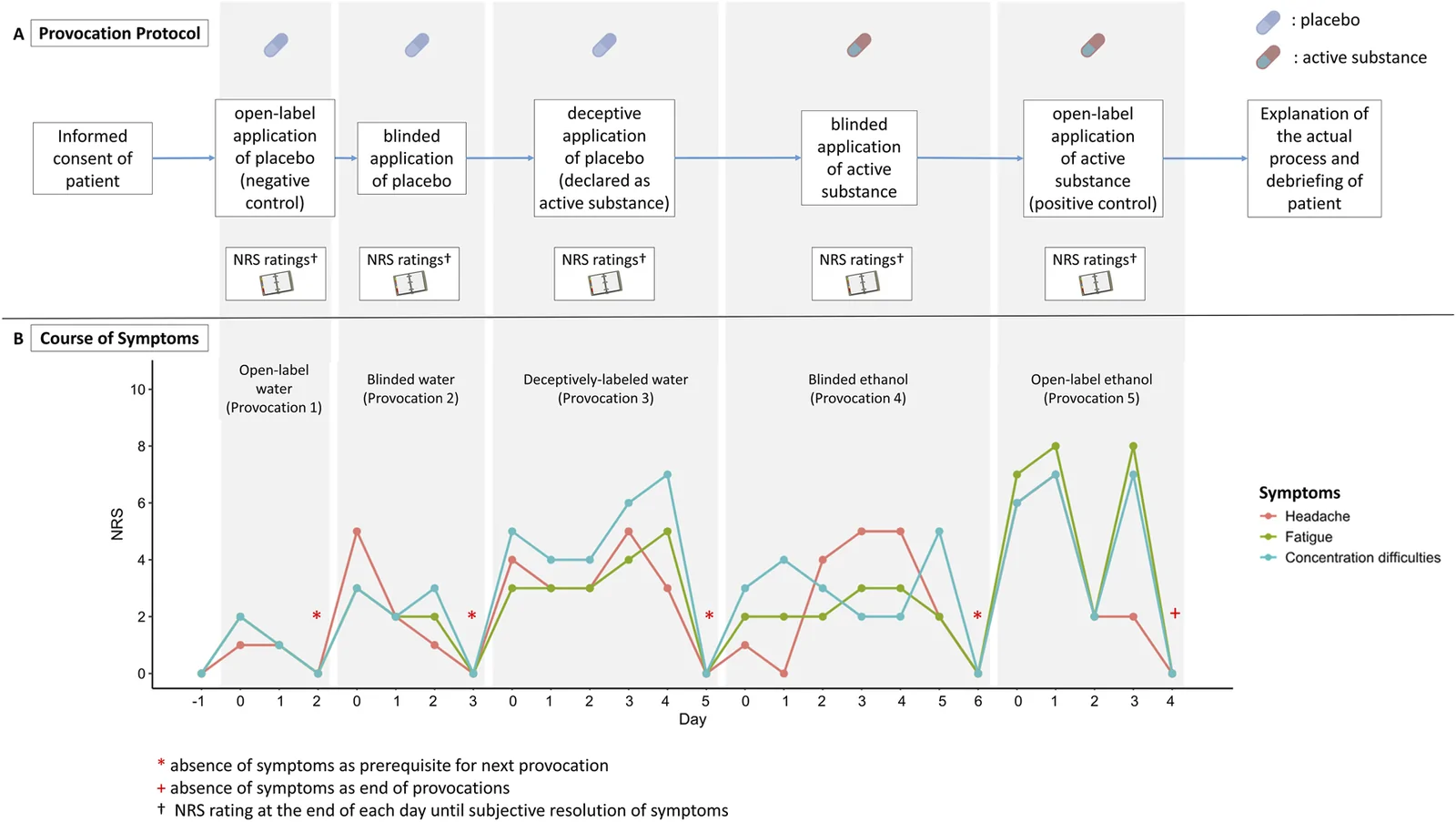
Course of symptoms in relation to type of provocation. **(A)** Schematic illustration of the *Bonn Provocation Protocol* consisting of five provocation sessions. **(** **B)** Time course of individual symptom intensity (for headache, fatigue, concentration difficulties) on the NRS scale following each provocation. The duration of the observation periods differ across provocation sessions, reflecting the variable duration until subjective resolution of symptoms as prerequisite for the next provocation. Source: own illustration.

The patient is led to believe that the order of exposure has been randomly decided, even though it has not, for practical reasons and in order to optimize the methodological validity of the research to establish the nocebo effect. Instead, the protocol predetermines that the patient will initially undergo provocation sessions with the placebos. This sequence is intended to prevent mixing with effects that could result from prior intake of the active substance (verum), which could be particularly relevant for substances with potentially unblinding side effects. Furthermore, it eliminates the need for washout phases prior to the subsequent placebo administration and the need of a randomization process in the clinical routine where staff members usually do not have experience with these processes.

### 3.2 Proof of concept

#### 3.2.1 Case description

A 39-year-old male patient (BMI 24.5 kg/m^2^, non-smoker) presented to our Clinical Pharmacology Outpatient Department with a history of pronounced alcohol intolerance that had gradually developed since a complicated dental extraction 5 years ago, which was associated with the intake of several antibiotics and analgesics. The patient reported that his alcohol consumption had always been low, but in the meantime even minimal amounts of alcohol—such as one sip of beer or topical application of ethanol-containing agents on the skin—would provoke immediate symptoms resembling a “drunken state” including headache, fatigue, and cognitive impairment over several days. In contrast, a food diary revealed that unrecognized exposure to ethanol in sauces, fruits, fruit juices, and yeast pastry did not elicit any reaction, suggesting a discrepancy between expected and unrecognized exposure. As a result, the patient’s social and psychological burden was considerable; he reported withdrawal from social life due to the fear of accidental alcohol exposure and an inability to explain his condition to peers and colleagues. The patient reported a general fear of having a serious physical illness, adverse physical reactions, and the resulting inability to engage in professional or social life. He had no current pharmacotherapy and expressed a strong desire to clarify his problem, both for symptom management and psychosocial relief.

The patient had already undergone extensive laboratory testing, including assessments of liver and kidney function, endocrine status (corticotropic, thyrotropic, and ganadotropic axes), and allergy diagnostics, none of which identified an underlying pathology. His medical history included moderate depressive episodes, irritable bowel syndrome, and prior diagnosis of attention-deficit/hyperactivity disorder (ADHD).

Alcohol intolerance reported by the patient could not be explained by physiological, allergic, or metabolic mechanisms (Agarwal and Goedde, 1990). The patient’s heightened symptom awareness, combined with the experience of previous adverse reactions to drugs and comorbidities in which psychological factors are known to play a significant role, such as depression and irritable bowel syndrome, were considered relevant given their association with increased susceptibility to nocebo effects (Häuser et al., 2012; Enck and Klosterhalfen, 2021). Additionally, stress and anxiety are also discussed as contributing factors (Colloca and Barsky, 2020), so the experience of complicated tooth extraction could have acted as an additional trigger explaining the temporal relationship.

The patient was informed about the significance and mechanisms of nocebo effects using a positive language, and it was discussed with him that he might also be experiencing this effect. To clarify symptoms, the patient was offered a blinded provocation test.

#### 3.2.2 Intervention

Prior to this individually designed single-patient multi-crossover study (n = 1) the patient received both verbal and written information about the general nature of the test design, during which he would be exposed to various interventions, including the use of both water (placebo) and ethanol (active substance), that could potentially include intentional misinformation as a method of expectation manipulation. After discussing the risks and benefits, including temporary withholding of exact information to preserve diagnostic validity, the patient consented to participate in this single-subject, crossover clinical trial.

The five outpatient provocation sessions were conducted over 4 weeks. Rather than adhering to a fixed schedule, the timing of the provocation was adapted to allow for the complete resolution of symptoms following which the patient scheduled the subsequent appointment himself. Consequently, the duration of the observation periods differed across provocation sessions (session 1: 2 days, session 2: 3 days, session 3: 5 days, session 4: 5 days, session 5: 4 days). All provocations were conducted on different days in the morning between 9 and 11 a.m. in the same clinical room under similar ambient conditions (room temperature), with neutral lighting. In the present case, no specifications regarding behaviour, diet, fasting status, or sleep were imposed on the patient; such standardisation may, however, be advisable for other cases with other active substances. Each provocation involved the administration of either 2 mL of 4 °C 99% ethanol as the active substance (verum) or 2 mL 4 °C tap water as a placebo administered onto a 5 cm × 5 cm area of healthy dry skin of the popliteal fossa bend using a sterile pipette, left in contact for 2 min, and subsequently removed with a gauze compress. The cooling was intended to prevent any difference in the sensation of alcohol or water on the skin. The patient wore an FFP2 mask to blind him to the smell of evaporation.

After each provocation, the patient was instructed to complete a symptom diary before going to bed documenting the most severe symptoms experienced since the previous diary entry for headache, fatigue, and concentration difficulties as the characteristic symptom complex reported by the patient using a numeric rating scale (NRS; 0–10) until subjective resolution of symptoms for a minimum duration of 48 h (corresponding to at least two values from two consecutive days); the follow-up periods therefore vary in duration across provocation sessions. A full debriefing followed the completion of the provocation test, during which the true contents of each provocation were disclosed and the rationale of the study design was explained to the patient.

#### 3.2.3 Ethics

Following review of the diagnostic approach, with specific attention to the deceptive component, ethics approval was waived by the responsible Ethics Committee of the Medical Faculty of the University Bonn (ref. 2025–222-BO) as under German law an individualized diagnostic intervention of this kind—conducted within the framework of routine clinical care, although deviating from established clinical processes—does not require formal ethics committee approval. This also applies to the process of deception, which falls within the physician’s therapeutic discretion, as the patient was informed that mislabeling might be involved at some point during the protocol.

#### 3.2.4 Statistical analysis

Due to the single-case n-of-1 design and the repeated-measures nature of the daily symptom tracking classical assumptions of independence for hypothesis testing are not met. Therefore, data analysis was strictly descriptive. Mean and median NRS scores were calculated for each symptom category across all documented days per session. An overall mean was calculated by averaging across all three symptom categories to evaluate the overall symptom burden for each provocation, as they represented the characteristic symptom complex reported by the patient. To quantify the magnitude of differences between specific provocation conditions, we calculated non-parametric effect sizes (Cliff’s delta) alongside their 95% confidence intervals based on the pooled daily NRS scores. All statistical analyses and visualizations were performed using RStudio (v2026.01.0 + 392) and the “*ggpubr*” (v0.6.3) package.

## 4 Results

Before the start of each of the five provocations, the patient was subjectively free of symptoms (NRS = 0), which was a prerequisite for beginning the next provocation test. In the open-label provocation with water (provocation 1), the patient reported only minor symptoms (mean NRS rating averaged across all symptoms was 1.3, with a maximum 2, Table 1). During the blinded provocation with water (provocation 2), he noted mild headache and fatigue (mean NRS 2.6, maximum 5), with uncertainty about what had been applied. Provocation 3, where water was falsely labeled and declared as ethanol, produced strong reactions, including persistent headache, severe fatigue, and concentration difficulties (mean NRS 4.1, maximum 7). These symptoms were comparable to the symptoms he experienced after former ethanol exposure. In contrast, the administration of ethanol in the blinded condition (provocation 4) again resulted in mild symptoms (mean NRS 2.8, maximum 5), similar in severity to the blinded water condition. Open-label ethanol administration (provocation 5) elicited the strongest reaction. The patient reported an immediate onset of fatigue, followed by severe headache later that day (mean NRS 5.3, maximum 8), although with a delayed onset. These reactions closely resembled those seen in provocation 3, highlighting the role of expectations.

**TABLE 1:** Daily symptom intensity ratings during provocation testing.

​	Open-label water (provocation 1)	Blinded water (provocation 2)	Deceptively-labeled water (provocation 3)	Blinded ethanol (provocation 4)	Open-label ethanol (provocation 5)
Headache	​	​	​	​	​
Mean ± SD	1.0 ± 0.0	2.7 ± 2.8	3.6 ± 0.9	2.8 ± 2.1	4.3 ± 2.6
Median [range]	1 [1, 1]	2 [1, 5]	3 [3, 5]	3 [0, 5]	4 [2, 7]
Fatigue	​	​	​	​	​
Mean ± SD	1.5 ± 0.7	3.3 ± 0.6	3.6 ± 0.9	2.3 ± 0.5	6.3 ± 2.9
Median [range]	1.5 [1, 2]	2 [2, 3]	3 [3, 5]	2 [2, 3]	7.5 [2, 8]
Concentration difficulties	​	​	​	​	​
Mean ± SD	1.5 ± 0.7	2.3 ± 0.6	5.2 ± 1.3	3.2 ± 1.2	5.5 ± 2.3
Median [range]	1.5 [1, 2]	2 [2, 3]	5 [4, 7]	3 [2, 5]	6.5 [2, 8]
Average across all symptoms	​	​	​	​	​
Mean ± SD	1.3 ± 0.5	2.6 ± 1.1	4.1 ± 1.2	2.8 ± 1.4	5.3 ± 2.5
Median [range]	1 [1, 2]	2 [1, 5]	4 [3, 7]	2.5 [0, 5]	6.5 [2, 8]
Observation period (days)	2	3	5	5	4
Symptom onset (day)*	0	0	0	0	0
Time to symptom resolution (days)	2	3	5	6	4
Peak NRS of most severe symptom (day)	2	5	7	5	8
Cliff’s δ vs. provocation 3	1.0 [95% CI: NA]	0.7 [95% CI: 0.20, 0.91]	n. a	0.54 [95% CI: 0.16, 0.78]	0.28 [95% CI: −0.26, 0.69]

Subjective symptoms of headache, fatigue, and concentration difficulties reported by the subject as scores of the numeric rating scale (NRS; 0–10) for each provocation session and the values averaged across all symptoms. The number of documented values/days corresponds to the duration of symptoms.

SD, standard deviation.

* Day 0 = Day of provocation.

Source: Own data.

Descriptive analysis of the magnitude of differences between provocation conditions using non-parametric effect sizes (Cliff’s Delta) revealed a strong effect when comparing the deceptively-labeled placebo administration to the open-label water administration (complete non-overlap with δ = 1.00; CI not estimable at complete separation), as well as to the blinded administrations of both water (δ = 0.70 [95% CI: 0.20, 0.91]) and ethanol (δ = 0.54 [95% CI: 0.16, 0.78]). By contrast, the difference between the deceptively-labeled placebo and the open-label ethanol condition was small (δ = 0.28 [95% CI: −0.26, 0.69]). Most importantly, no substantial difference was observed between the blinded administration of both water and ethanol (δ = 0.10 [95% CI: −0.34, 0.50]). The comparison between blinded ethanol and open-label ethanol demonstrated a moderate effect (δ = 0.54 [95% CI: 0.04, 0.82]). The detailed time course and data of the individual symptoms is presented in Figure 1B and summarized in Figure 2.

**FIGURE 2:**
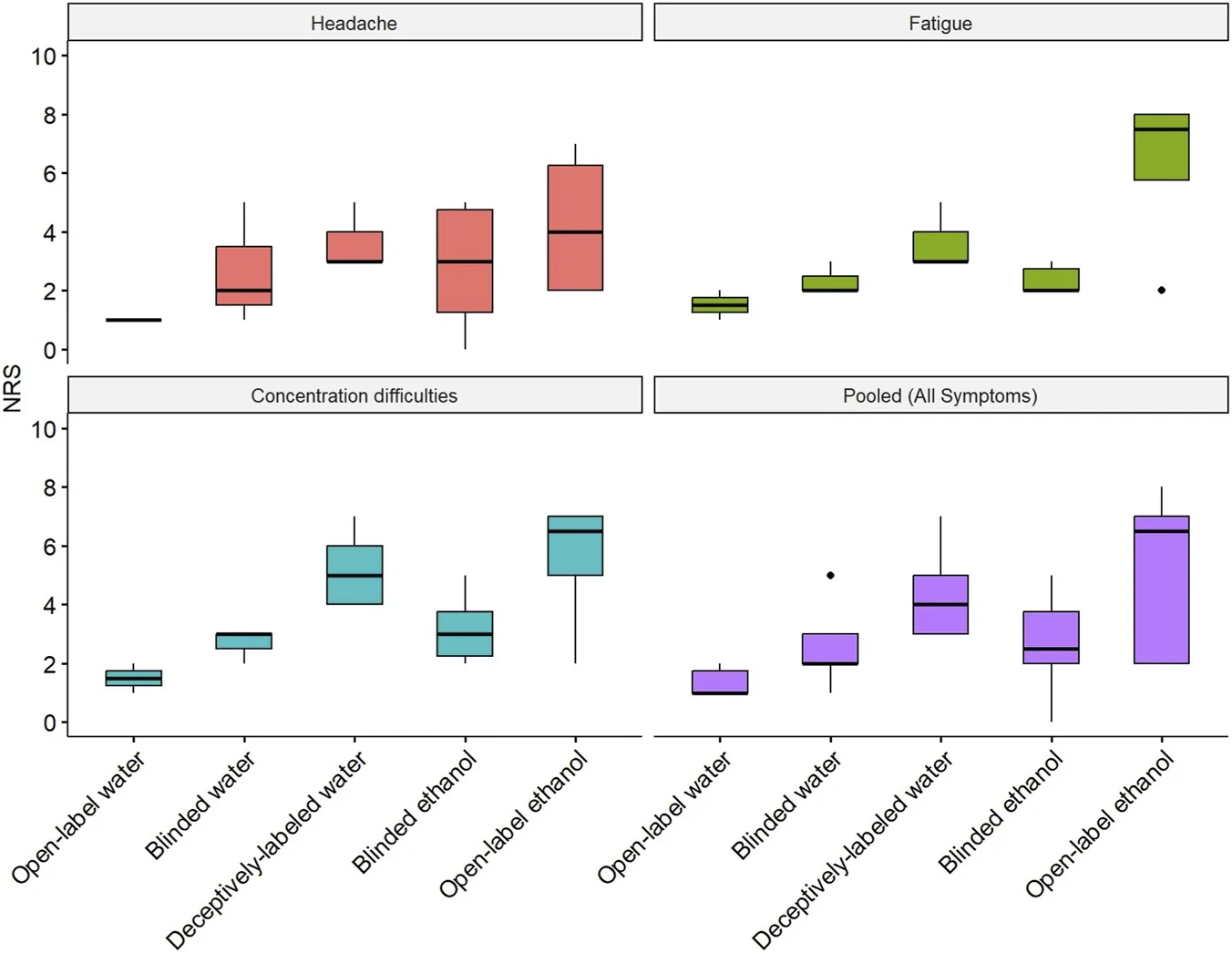
Symptom distribution across provocations. Boxplots of daily symptom intensity ratings after provocation testing separated for each symptom: Numeric Rating Scale (NRS) scores (0–10) recorded for the most severe headache, fatigue, and concentration difficulties by the patient each day after each provocation session. Source: own illustration.

Upon learning the true nature of the substances used during the debriefing, the patient expressed surprise and reported a sense of relief that the testing helped uncover these underlying connections. The patient denied experiencing any psychological distress resulting from the procedure or from its disclosure. During a follow-up period of 1 year, the patient reported a subjective improvement in his overall wellbeing and the renewed ability to participate in social activities. He had cautiously resumed alcohol consumption without experiencing comparable symptoms.

## 5 Discussion

This report illustrates the initial proof-of-concept of a novel modified crossover balanced placebo design (*Bonn Provocation Protocol*) to identify adverse effects potentially attributable to nocebo reactions in routine clinical practice. To our knowledge, this specific design has not previously been described and it is the first report of applying such a design for diagnostic use in routine clinical practice rather than for research purposes. It describes the procedure of a structured reproducible protocol for clinicians managing patients with unexplained drug intolerances. The findings do not provide evidence of diagnostic accuracy or generalizability. Rather they demonstrate the general feasibility and an initial proof-of-concept description of the approach raising the hypothesis that methods hitherto confined to experimental research can be successfully transferred to real-world clinical settings. In comparison to our previously published statin rechallenge protocol (Büttner et al., 2025), this design represents an extension that incorporates additional expectation manipulations within the same subject. Unlike prior studies that tested drug and placebo effects (Hammami et al., 2010; Enck et al., 2011; Lund et al., 2014; Hammami et al., 2016), the distinctive feature of our design is the combination of placebo and verum administration under open and blinded conditions, paired with either correct or intentionally misleading information, *all within the same subject*, which could be termed a “*mixed open-blinded crossover balanced placebo design*”. The additional use of a deceptively labeled administration of the active substance would make the variants of provocations complete. However, this is dispensable in the context of nocebo diagnostics as this would only analyze the influence of expectations on specific pharmacological effects rather than identify nocebo-mediated symptoms. The use of both open-label and deceptive conditions should make it possible to differentiate between pharmacological and psychological (expectancy-driven) effects in this individual patient. The variation in the patient’s reactions to water across provocations one to three, from no symptoms under open-label administration to moderate symptoms under blinded administration, and severe symptoms following mislabeled water, suggests that symptom expression in this patient may have been primarily driven by expectations rather than pharmacological effects. Furthermore, the similarity in symptom severity between both blinded provocations (water and ethanol) as well as both administrations labeled as ethanol (water and ethanol) is consistent with the hypothesis that expectation effects may override true pharmacological inputs entirely, even going beyond the non-additive model of placebo and nocebo effects (Enck et al., 2013). The within-subject crossover structure allowed a direct, internally valid comparison of pharmacological and expectancy-driven responses as the patient served as his own control across all five conditions yielding potentially informative findings with therapeutic consequences. This is consistent with the core principle of n-of-1 trials, in which repeated within-subject comparisons serve to clarify individual cause-and-effect relationships. This approach is particularly well-suited to expectation-sensitive symptoms, which may manifest differently in each patient and are therefore not amenable to generalization from group-level data (Mijnster et al., 2025).

Overall, these key observations in our patient are consistent with nocebo effects rather than a true pharmacological intolerance, suggesting that the clinical diagnosis of alcohol intolerance actually was not appropriate and could be unmasked by the provocation tests. This clinical assessment–a question that cannot be answered by standard diagnostic approaches—allowed the patient to reflect on and reinterpret his symptoms leading to subjective improvement in his overall wellbeing and the renewed ability to participate in social activities. However, we emphasize that these interpretations are based on a single uncontrolled case in the specific context of topical alcohol as the test substance preventing generalization. Alternative explanations cannot be definitively ruled out. The lack of observer-blinding may have introduced observer bias and bias of subtle cues influencing the patient’s symptom reporting. The lack of randomization and the fixed session order prevent assessment of potential order effects. However, the fixed sequence, with placebo administered before the active substance, was itself intended to minimize bias. Earlier administration of the active substance—whether planned or randomized—would have entailed an increased risk of carry-over effects, learning effects, and expectancy drift, given the pharmacological exposure and the experience of genuine adverse effects potentially arising from it. Finally, it is known that expectations may change dynamically during a blinded study as a result of trial experience and (un)blinding (Huneke et al., 2025). Therefore, repeated exposure to our provocation conditions may itself have modified expectations over the course of the protocol. This may be addressed by future studies through randomized or counterbalanced designs. However, the fixed order eliminates the need for washout phases prior to subsequent placebo administration and the need of a randomization process, both of which would complicate the implementation in routine clinical practice.

Furthermore, all outcomes relied on patient self-report without objective physiological measurements, spontaneous symptom fluctuations may have contributed to the observed patterns and symptom assessment relied exclusively on unidimensional numeric rating scales. The NRS is well validated for pain (Ferreira-Valente et al., 2011) and is applied specifically to headache. For fatigue, single-item ratings have not been validated to the same extent, yet the unidimensional NRS is frequently used (Chuang et al., 2015). For concentration difficulties, dedicated instruments are available (Saffer et al., 2015; Waldron-Perrine et al., 2019; Gass et al., 2021). We nevertheless applied the NRS across all three domains as even without formal validation, it can reasonably be assumed that it reflected the patient’s subjective assessment of symptom severity primarily relevant for this relatively straightforward clinical question. Furthermore, as suspected adverse drug reactions in clinical practice may involve a wide, largely unpredictable range of complaints, each would require a different validated assessment instrument with questionable additional benefit. Therefore, these conditions should be regarded as appropriate for such clinical diagnostic questions to ensure practical implementation in routine clinical care.

Although the patient’s presentation represents a rare and specific clinical scenario, it was an ideal case to demonstrate the general approach while being highly illustrative at the same time. These findings are consistent with the powerful influence of reinforced expectations as important determinants of symptom expression (Colloca and Miller, 2011) and are also in line with the existing literature, demonstrating that negative expectations alone can lead to subjective symptoms (Barsky et al., 2002; Enck et al., 2011; Rooney et al., 2024). It is well known that a counteracting expectation can attenuate, abolish, or even reverse the pharmacological effect of an active substance—and conversely, that a positive expectation can produce an effect even in the absence of an active drug (Hammami et al., 2010; Enck et al., 2011; Bingel et al., 2011). This phenomenon can be termed as a “*reverse expectation effect.*” This effect also explains why responses to active substances are attenuated when trial participants suspect they are receiving placebo, and conversely why larger effects may be observed in poorly blinded studies (Huneke et al., 2025; Taylor et al., 2026). For this provocation protocol we have now deliberately exploited this phenomenon for clinical diagnostics. As a conceptual complement to the existing terms *nocebo* and *drucebo* (Penson et al., 2018) used in related contexts, we propose the term “*deceptebo*” (derived from “deception”) to describe the experience of adverse effects after administration of mislabeled placebo triggering the belief that an active drug has been administered. No established term for this specific phenomenon exists so far. This conceptual proposal and the appropriateness and utility of this term requires validation and should be evaluated in future research.

A simple structured single-blind placebo provocation test may be diagnostically sufficient (Büttner et al., 2025) in many cases. However, the additional active manipulation of patient expectations through the deceptive administration of placebo, in addition to open-label and blinded placebo conditions, facilitates the distinction between suspected adverse drug reactions and nocebo effects. Specifically, the deceptively labeled placebo condition is the only component that directly tests the causal contribution of the belief that ethanol had been administered. The inclusion of deception elements in clinical testing requires careful justification and transparent consent. In our case, the patient was informed that certain information would be temporarily withheld or intentionally misleading without disclosing the specific nature or details of the deception, and he consented to this approach. Most analyses assume that deception can be acceptable, provided that the subject is generally informed about the process and that it does not conceal any risks (Wendler and Miller, 2004; Matvey et al., 2025). Our approach corresponds to the concept of “authorized deception” proposed by Miller et al. (Miller et al., 2005) established in placebo research (Hammami et al., 2010; Enck et al., 2011). Informing patients upfront that some information will be temporarily withheld—without revealing specifics—has been shown not to compromise the validity of the procedure or the therapeutic relationship (Hammami et al., 2010; 2016), does not impair recruitment or retention, and is preferred by the majority of participants over non-disclosure (Martin and Katz, 2010). Notably, we were able to exclude significant physical risks to the patient in this specific case, as dermal absorption of a hand disinfectant could be confidently ruled out as causing systemic effects. Furthermore, the administration of a placebo under deceptive labeling eliminates specific pharmacological risks associated with the mislabeled substance itself. In cases involving the deceptive use of active medications, particular attention should be paid to inform patients about medically relevant side effects (Burke et al., 2026). Furthermore, it should be considered that the process of mislabeling and deception may not be legally permissible in all jurisdictions. For the protocol, we obtained a waiver from our ethics committee specifically for this case, which does not imply that similar deceptive protocols automatically qualify for exemption. Clinicians wishing to implement this protocol should therefore consult applicable legal and ethical requirements in their jurisdiction (Richard et al., 2025).

The present case involved a topical provocation, which is particularly amenable to blinding, as placebo and verum can be matched for volume and temperature and olfactory cues can be masked. Transfer to other routes of administration appears feasible but requires route-specific considerations. Oral provocation is most readily transferable, since identical-appearing capsules or overencapsulation are established practice in drug challenge testing (Khan et al., 2022) For injectable drugs, saline offers a straightforward placebo, but the invasiveness of repeated administrations raises the threshold for ethical justification (De Deyn and D’Hooge, 1996), and formulation-specific injection site reactions may compromise blinding. Biologics appear least suitable as delayed and protracted symptom onset impedes attribution to individual sessions, interrupted exposure entails a risk of immunogenicity, and their long half-lives preclude adequate wash-out within a crossover design.

Full debriefing is key to maintaining trust and ensuring a positive outcome. Following a review of the findings, the patient reported a substantial reduction in anxiety regarding alcohol exposure and resumed certain social activities that improved his quality of life. The patient’s relief and gratitude following the improvement in wellbeing are consistent with the potential value of translating methods from nocebo research into clinical practice although findings from a single case cannot establish diagnostic utility or generalizability. Repeating the same provocation within this already demanding protocol to corroborate the results in this patient would, however, also be impractical and ethically difficult to justify. A five-period crossover design with multiple visits, direct clinician supervision throughout each provocation, repeated exposures, and the use of deception might be less suitable for routine clinical use because of the time and resources required. For a range of patients and complaints, a structured single-blind placebo provocation is likely to be diagnostically sufficient in general practice (Büttner et al., 2025). To simplify the practical administration of our design, it would also be conceivable to omit the two open-label administrations of placebo and active substance at the beginning and the end of the approach, probably without compromising its diagnostic value. We are currently investigating a similar approach in a clinical trial. The practical implementation could be based on the process described recently (Büttner et al., 2025). Where further investigation is required, referral to a specialized center for the full balanced placebo design may be more appropriate. Implementation outside specialised clinical pharmacology centers and across different care settings warrants further investigation.

Taken together, this report describes the approach and an illustrative case of a single patient of how nocebo effects can be actively addressed in clinical practice. The case illustrates that the intervention may inform clinical decision-making in this individual case as it allowed the patient to regain confidence. The results are consistent with the idea of provocation testing in differentiating true pharmacological adverse effects from nocebo-driven symptoms in patients with unexplained symptoms of intolerance. However, it should not be understood as an attempt to establish generalizability. Controlled studies in larger patient cohorts would be necessary to systematically evaluate the robustness of the approach beyond hypothesis generation. However, several factors complicate a systematic investigation: the symptoms the patients present with and the triggering substances are highly diverse, and the absence of a definitive diagnostic criterion for nocebo effects that would allow reliable patient selection—since enrolling patients with only suspected nocebo effects would inherently compromise the validity of the provocation test results. From a clinical perspective, however, all provocation sessions in routine practice would by definition be conducted in a single patient; a result such as the one described would nonetheless substantiate a suspicion of nonspecific adverse effects and directly inform the further management of that patient.

## Data Availability

The raw data supporting the conclusions of this article will be made available by the authors, without undue reservation.
